# VHDL Descriptions for the FPGA Implementation of PWL-Function-Based Multi-Scroll Chaotic Oscillators

**DOI:** 10.1371/journal.pone.0168300

**Published:** 2016-12-20

**Authors:** Esteban Tlelo-Cuautle, Antonio de Jesus Quintas-Valles, Luis Gerardo de la Fraga, Jose de Jesus Rangel-Magdaleno

**Affiliations:** 1 Department of Electronics, INAOE, Tonantzintla, Puebla, Mexico; 2 Department of Computer Science, CINVESTAV, Zacatenco, Mexico City, Mexico; Lanzhou University of Technology, CHINA

## Abstract

Nowadays, chaos generators are an attractive field for research and the challenge is their realization for the development of engineering applications. From more than three decades ago, chaotic oscillators have been designed using discrete electronic devices, very few with integrated circuit technology, and in this work we propose the use of field-programmable gate arrays (FPGAs) for fast prototyping. FPGA-based applications require that one be expert on programming with very-high-speed integrated circuits hardware description language (VHDL). In this manner, we detail the VHDL descriptions of chaos generators for fast prototyping from high-level programming using Python. The cases of study are three kinds of chaos generators based on piecewise-linear (PWL) functions that can be systematically augmented to generate even and odd number of scrolls. We introduce new algorithms for the VHDL description of PWL functions like saturated functions series, negative slopes and sawtooth. The generated VHDL-code is portable, reusable and open source to be synthesized in an FPGA. Finally, we show experimental results for observing 2, 10 and 30-scroll attractors.

## 1 Introduction

Chaos theory deals with nonlinear and complex dynamic behavior that is associated to unpredictable phenomena. The main characteristic is that small changes in the initial conditions lead to drastic changes in the results. It is deterministic because one knows its model parameters and it is unpredictable because one does not know the evolution of the trajectories, and then one cannot predict its behavior.

Three decades ago, the author in [1] introduced an extremely simple autonomous circuit that generates chaotic behavior. It was known as Chua’s circuit with the advantage of including only one nonlinear element composed of a piecewise-linear (PWL) resistor. If it has 3-segments, it can generate the double-scroll attractor, as confirmed in [2]. Ten years later, the popularity of Chua’s circuit was summarized in [3], where it is mentioned that more than 200 papers were published at that time since its inception in 1984. The important milestone was the fabrication of an integrated circuit for Chua’s circuit to observe the double-scroll attractor. In addition, it was demonstrated that Chua’s circuit can be easily controlled from a chaotic regime to a prescribed periodic or constant orbit, or it can be synchronized with two or more identical Chua’s circuits operating in chaotic regime. Nowadays, it is very well-known that chaos generators are quite useful to develop applications in robotics, noise generators, random number generators, chaotic secure communications [4–7], and so on [8–15].

Chua’s circuit has been the most extensively studied chaos generator. For instance, the PWL resistor was modified in [16] with additional break points to generate *n*-double scrolls (*n* = 1, 2, 3, 4, …). It was a generalization of Chua’s circuit where the 1-double scroll corresponds to the classical double scroll one. Generating more than 2-scrolls was the challenge after Chua’s circuit. In 2004 [17] a systematic approach based on saturated function series was introduced to generate multi-scroll chaotic attractors from a three-dimensional linear autonomous system. That work also introduced the generation of multi-scrolls in 1-direction (1-D), 2-D and 3-D, thus generating 1-D *n*-scroll, 2-D *n* × *m*-grid scroll, and 3-D *n* × *m* × *l*-grid scroll chaotic attractors. The experimental verification of those chaotic attractors was reported in [18], where the authors provided guidelines for analog hardware implementation. 4-D attractors are also possible, as introduced in [19]. From more than 3 decades ago, the majority of chaos generators have been realized using discrete electronic devices and very few using integrated circuit technology [20]. Recently, chaos generators have been implemented using field-programmable gate arrays (FPGAs) [4, 21], for fast prototyping and also to tune fractional coefficient values, which are difficult when using traditional operational amplifiers [20]. However, the hardware realization depends on the numerical method that discretizes the dynamical equations [21], which remains as a challenge to implement robust chaos generators.

As one can infer, multi-scroll chaotic oscillators have more complex behavior than traditional double-scroll ones. They are quite useful for engineering applications [4], and they can easily be implemented using FPGAs [21], which provide flexibility and capability of being reprogrammed/configured. In fact, it is said that configurability for engineering applications makes FPGA very crucial in initial stages for any embedded project. In this manner, we introduce a systematic approach for the VHDL description of PWL functions for the FPGA implementation of chaos generators based on saturated function series, negative slopes and sawtooth function. We use Python as a high-level description mechanism [22], to describe hardware modules of three dimensional chaotic oscillators. That way, from mathematical models we show how to describe them in VHDL-code [23–25], which is ready to be synthesized into an FPGA. This is our contribution for fast prototyping [26, 27], and can help as a computer-aided design tool [24], for the FPGA implementation of multi-scroll chaotic oscillators.

The rest of the article is organized as follows: Sect. 2 details three kinds of chaos generators. Their PWL functions to generate multi-scroll chaotic attractors are described in Sect. 3. From those descriptions we introduce equations to calculate the number of hardware blocks that will be created like VHDL code, as shown in Sect. 4. Since FPGA realizations require the use of a numerical method, sub-section 4.2 shows that by using Forward Euler (FE) and fourth-order Runge-Kutta, one gets similar values of the maximum Lyapunov exponent (MLE), thus FE is good enough to observe chaotic attractors and also it consumes the lowest FPGA resources than other methods. Section 5 shows experimental results, and Sect. 6 summarizes the conclusions.

## 2 Multi-scroll chaotic oscillators

Three kinds of 3-dimensional multi-scroll chaotic oscillators are described herein. All of them are based on PWL functions that can be designed in a systematic way to generate odd or even number of scrolls. The following subsections show new ways to model PWL functions like saturated function series, negative slopes and sawtooth one. All these PWL functions can be implemented using comparators, which will be useful for the VHDL descriptions of chaos generators, as shown in Sect. 4.

### 2.1 Chaotic oscillator based on saturated function series

**Fig 1:**
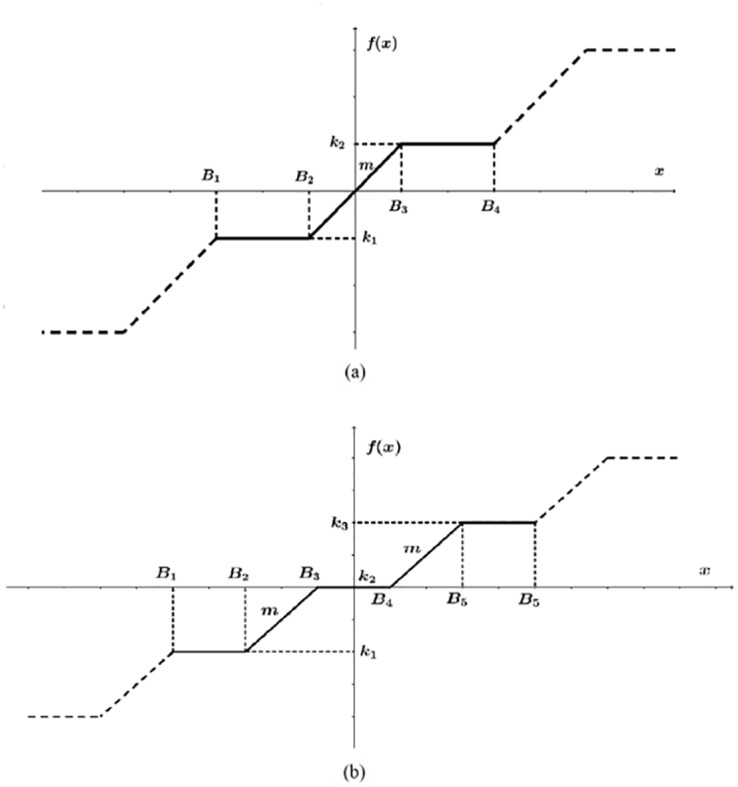
PWL function based on saturated function series to generate. (a) even and (b) odd number of scrolls.

Eq (1) describes the 3-dimensional multi-scroll chaotic oscillator based on the PWL function *f*(*x*) described by Eq (2). The state variables are *x*, *y* and *z*, and they are multiplied by four coefficients: *a*, *b*, *c* and *d*_1_. Those coefficient values can be in the range [0, 1], and one must decide how many places to use for the integer and fractional parts [4, 21]. The PWL function can be associated to comparators, since it is a saturated function series. From Fig 1, one can identify the parameters: saturation levels *k*_*i*_, break points *B*_*j*_ and slope *m*. These three parameters can be augmented according to the number of scrolls *n* being generated. The PWL function in continuous lines shown in Fig 1(a) can be described by Eq (2), which can be augmented as sketched by the dashed lines to generate even number of scrolls. In a similar manner, Fig 1(b) has parameters *k*_*i*_, *B*_*j*_ and *m*, but the continuous line has 3 saturation levels (*k*_1_, *k*_2_, *k*_3_) to generate 3-scrolls, then the PWL function can be augmented as sketched by the dashed lines to generate odd number of scrolls, and the mathematical description is like in Eq (3), where the PWL function will have *n* number of saturation levels and *n* − 1 slopes *m*.

$$\begin{array}{ccc} \dot{x} & = & y\\ \dot{y} & = & z\\ \dot{z} & = & -ax-by-cz+d_{1}f\left(x\right) \end{array}$$ (1)

$$\begin{array}{c} f\left(x\right)=\{\begin{array}{cc} \ldots & \\ k_{1}, & \text{if}\;B_{1}<x<B_{2}\\ mx, & \text{if}\;B_{2}\leq x\leq B_{3}\\ k_{2}, & \text{if}\;B_{3}<x<B_{4}\\ \ldots \end{array} \end{array}$$ (2)

$$\begin{array}{c} f\left(x\right)=\{\begin{array}{cc} \ldots & \\ k_{1}, & \text{if}\;B_{1}<x<B_{2}\\ m\left(x-\frac{B_{2}+B_{3}}{2}\right)-\frac{k_{1}+k_{2}}{2}, & \text{if}\;B_{2}\leq x\leq B_{3}\\ k_{2}, & \text{if}\;B_{3}<x<B_{4}\\ m\left(x-\frac{B_{4}+B_{5}}{2}\right)-\frac{k_{2}+k_{3}}{2}, & \text{if}\;B_{4}\leq x\leq B_{5}\\ k_{3}, & \text{if}\;B_{5}<x<B_{6}\\ \ldots \end{array} \end{array}$$ (3)

### 2.2 Chua’s circuit based on negative slopes

**Fig 2:**
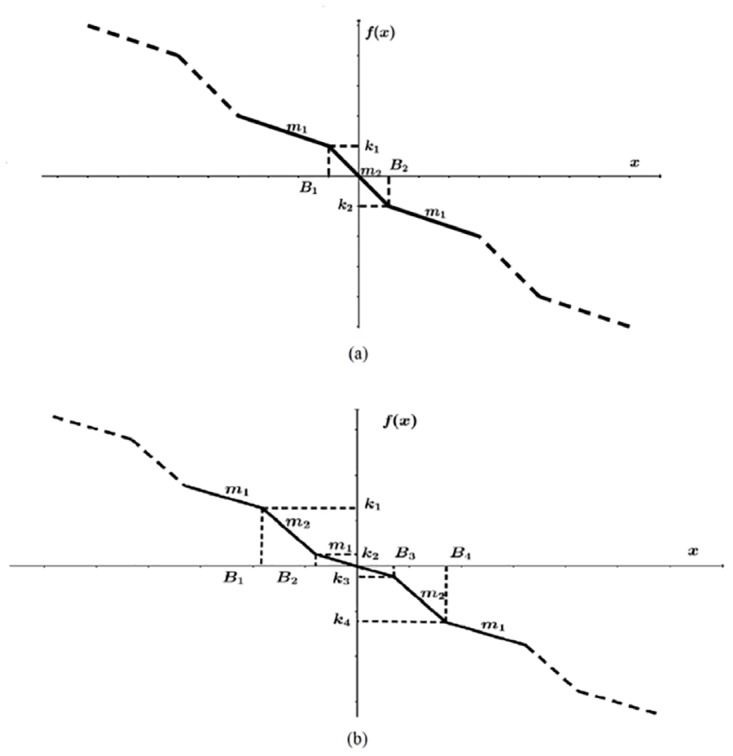
PWL function based on negative slopes to generate. (a) even and (b) odd number of scrolls.

Chua’s circuit has also three state variables: *x*, *y* and *z*; as described by Eq (4), and their coefficients are *α*, *β* and *γ*. From Fig 2, one can identify the parameters: amplitude *k*_*i*_, break points *B*_*j*_ and slopes *m*_*e*_. These three parameters can be augmented according to the number of scrolls *n* being generated. The PWL function in continuous lines shown in Fig 2(a) can be described by Eq (5), which can be augmented as sketched by the dashed lines to generate even number of scrolls. Fig 2(b) has also parameters *k*_*i*_, *B*_*j*_ and *m*_*e*_, but the continuous line has more segments than Fig 2(a) to generate 3-scrolls, then the PWL function can be augmented as sketched by the dashed lines to generate odd number of scrolls, and the mathematical description is like in Eq (6).

$$\begin{array}{ccc} \dot{x} & = & \alpha (y-x-f(x))\\ \dot{y} & = & \gamma (x-y+z)\\ \dot{z} & = & -\beta y \end{array}$$ (4)

$$\begin{array}{c} f\left(x\right)=\{\begin{array}{cc} \ldots & \\ m_{1}\left(x-B_{1}\right)+k_{1}, & \text{if}\;x<B_{1}\\ m_{2}x, & \text{if}\;B_{1}\leq x\leq B_{2}\\ m_{1}\left(x-B_{2}\right)+k_{2}, & \text{if}\;x>B_{2}\\ \ldots \end{array} \end{array}$$ (5)

$$\begin{array}{c} f\left(x\right)=\{\begin{array}{cc} \ldots & \\ m_{1}\left(x-B_{1}\right)+k_{1}, & \text{if}\;x<B_{1}\\ m_{2}\left(x-B_{2}\right)+k_{2}, & \text{if}\;B_{1}\leq x\leq B_{2}\\ m_{1}x, & \text{if}\;B_{2}<x<B_{3}\\ m_{2}\left(x-B_{3}\right)+k_{3}, & \text{if}\;B_{3}\leq x\leq B_{4}\\ m_{1}\left(x-B_{4}\right)+k_{4}, & \text{if}\;x>B_{4}\\ \ldots \end{array} \end{array}$$ (6)

### 2.3 Chua’s circuit based on sawtooth function

**Fig 3:**
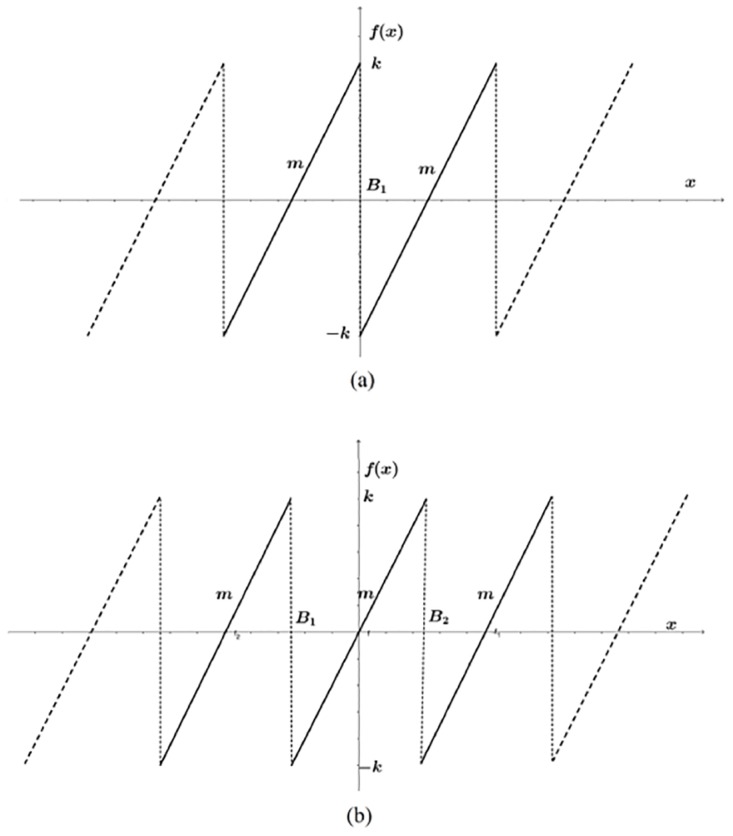
PWL function based on sawtooth function to generate. (a) even and (b) odd number of scrolls.

Chua’s circuit can also be implemented using a sawtooth function, and described by the three state variables *x*, *y* and *z*, given in Eq (7), and it also has three coefficients: *α*, *β* and *γ*. From Fig 3, one can identify the parameters: amplitude *k*_*i*_, break points *B*_*j*_ and slopes *m*. These three parameters can be augmented according to the number of scrolls *n* being generated. The PWL function in continuous lines shown in Fig 3(a) can be described by Eq (8), which can be augmented as sketched by the dashed lines to generate even number of scrolls. Fig 3(b) has 3 continuous lines to generate 3-scrolls, then the PWL function can be augmented as sketched by the dashed lines to generate odd number of scrolls, and the mathematical description is like in Eq (9). The PWL function will always have *n* − 1 number of break points *B*_*i*_, *n* number of slopes *m*, and amplitude ±*k*.

$$\begin{array}{ccc} \dot{x} & = & \alpha (y-f(x))\\ \dot{y} & = & \gamma (x-y+z)\\ \dot{z} & = & -\beta y \end{array}$$ (7)

$$\begin{array}{c} f\left(x\right)=\{\begin{array}{cc} \ldots & \\ m(x-B_{1})-k, & \text{if}\;x<B_{1}\\ m(x-B_{1})+k, & \text{if}\;x>B_{1}\\ \ldots \end{array} \end{array}$$ (8)

$$\begin{array}{c} f\left(x\right)=\{\begin{array}{cc} \ldots & \\ m(x-B_{1})-k, & \text{if}\;x<B_{1}\\ mx, & \text{if}\;B_{1}<x<B_{2}\\ m(x-B_{2})+k, & \text{if}\;x>B_{2}\\ \ldots \end{array} \end{array}$$ (9)

## 3 Simulating multi-scroll chaotic oscillators based on PWL functions

This section details the pseudocode for the three multi-scroll chaotic oscillators shown above. We describe the PWL functions with the main purpose of transforming the mathematical model to VHDL-code, as highlighted in the next section. For instance, as already shown in [4, 21], the mathematical descriptions in Eqs (1), (4) and (7) can be solved by numerical methods like Forward Euler, Runge-Kutta, and so on. The discretized equations from Eqs (1), (4) and (7), by applying Forward Euler, are given by Eqs (10), (11) and (12), respectively.

$$\begin{array}{ccc} x[k+1] & = & x[k]+hy[k]\\ y[k+1] & = & y[k]+hz[k]\\ z[k+1] & = & z\left[k\right]+h(-ax\left[k\right]-by\left[k\right]-cz\left[k\right]+d_{1}f\left(x\left[k\right]\right)) \end{array}$$ (10)

$$\begin{array}{ccc} x[k+1] & = & x[k]+h\alpha (y[k]-x[k]-f(x[k]))\\ y[k+1] & = & y[k]+h\gamma (x[k]-y[k]+z[k])\\ z[k+1] & = & z[k]-h\beta y[k] \end{array}$$ (11)

$$\begin{array}{ccc} x[k+1] & = & x[k]+h\alpha (y[k]-f(x[k]))\\ y[k+1] & = & y[k]+h\gamma (x[k]-y[k]+z[k])\\ z[k+1] & = & z[k]-h\beta y[k] \end{array}$$ (12)

The oscillators and their associated PWL functions can be programmed from the following pseudocodes to generate double-scroll attractors using Eqs (2), (5) and (8).

### 3.1 Oscillator based on saturated function series

To generate even and odd number of scrolls from Eq (1), *f*(*x*) is sketched in Fig 1(a) and 1(b), respectively. As one can infer, the PWL descriptions based on saturated function series and modeled in Eqs (2) and (3) can be extended according to the number of scrolls being generated. Algorithm 1 highlights the form in which the double-scroll chaotic attractor is simulated using Eq (2). The steps are executed as follows:

1. Loop to iterate st times
2. Initializes parameter p = 0
3. Initializes parameter q = 0
4. State variable xn[j] is updated to x1 + h∗y1, where x1 and y1 are initial conditions and h is the step size
5. State variable yn[j] is updated to y1 + h∗z1, where y1 and z1 are initial conditions and h is the step size
6. Verifies if variable x1 is between the break points values B[q] and B[q+1]. If it is satisfied then
7. Variable d1PWL is equated to d1∗k[p], where d1 corresponds to coefficient *d*_1_ in Eq (1) and k[p] to a saturation level in Eq (2)
8. Loop to iterate 2(*n* − 1) times, where *n* is the number of scrolls being generated
9. Verifies if x1 is between the break points B[q+1] and B[q+2]. If it is satisfied then
10. d1PWL is equated to d1∗(((k[p+1]-k[p]) /(B[q+2]-B[q+1]))∗(x1-((B[q+1]+B[q+2]) /2))+((k[p+1]+k[p])/2))
11. If step 9 is not satisfied then
12. d1PWL is equated to d1∗k[p+1]
13. Increases q by 2
14. Increases p by 1
15. State variable zn[j] is updated to z1+h(-a∗x1 − b∗y1 − c∗z1 + d1PWL), where a, b, c are coefficients in Eq (1)
16. State variable x1 is updated to the next iteration
17. State variable y1 is updated to the next iteration
18. State variable x1 is updated to the next iteration
19. Index j from step 1 is incremented to the next iteration, until st is accomplished.

**Algorithm 1.** Generating 2-scrolls using saturated function series

1   while j<st:

2    p = 0

3    q = 0

4    xn.insert(j, x1+h∗y1)

5    yn.insert(j, y1+h∗z1)

6    if x1 > B[q] and x1 < B[q+1]:

7     d1PWL = d1∗k[p]

8    while q < 2∗(n-1):

9     if x1 >= B[q+1] and x1 <= B[q+2]:

10      d1PWL = d1∗(((k[p+1]-k[p])/(B[q+2]-B[q+1]))∗(x1-((B[q+1]+B[q+2])/2))+((k[p+1]+k[p])/2))

11     elif x1 > B[q+2] and x1 < B[q+3]:

12      d1PWL = d1∗k[p+1]

13     q = q+2

14     p = p+1

15    zn.insert(j,z1+h∗(-a∗x1 − b∗y1 − c∗z1 + d1PWL))

16    x1 = xn[j]

17    y1 = yn[j]

18    z1 = zn[j]

19    j = j+1

### 3.2 Oscillator based on negative slopes

For Chua’s circuit based on negative slopes, to generate even and odd number of scrolls from Eq (4), *f*(*x*) must be described as shown in Eqs (5) and (6), respectively. Algorithm 2 highlights the form in which the double-scroll chaotic attractor is simulated using Eq (5). The steps are executed as follows:

1. Loop to iterate st times
2. Initializes parameter j = 0
3. Initializes parameter j1 = 0
4. Verifies if x in *f*(*x*) is lower than the break point B[0]. If it is satisfied then
5. Variable PWL is equated to m∗(x-B[0])+k[0]), where m is the slope, B[0] the break point and k[0] the amplitude
6. Loop to iterate (2*n* − 3) times, where *n* is the number of scrolls being generated
7. Verifies if x in *f*(*x*) is between the break points B[j] and B[j+1]. If it is satisfied then
8. PWL is equated to m[j]∗(x-B[j+1-j1])+k[j+1-j1]
9. Verifies if j is equal to n-3. If it is satisfied then
10. Index j is equated to j+2
11. Index j1 is equated to 1
12. Verifies if j fails the above then
13. Index j is equated to j+1
14. Verifies if x in *f*(*x*) is between the break points B[n-2] and B[n-1]. If it is satisfied then
15. PWL is equated to m[n-2]∗x, where m[n-2] correponds to slope *m*_1_ or *m*_2_ in Eqs (5) and (6)
16. Verifies if x is higher than B[2∗n-3]. If it is satisfied then
17. PWL is equated to m∗(x-B[2∗n-3])+k[2∗n-3]
18. State variable x[n] is updated to x+h∗(alpha∗(y-x-PWL)), where alpha is coefficient *α* in Eq (4), x and y are initial conditions, and h is the step size
19. State variable y[n] is updated to y+h∗gamma∗(x-y+z), where gamma is coefficient *γ* in Eq (4) and z is the initial condition
20. State variable z[n] is updated to z+h∗(-beta∗y), where beta is coefficient *β* in Eq (4)
21. State variable x is updated to the next iteration
22. State variable y is updated to the next iteration
23. State variable z is updated to the next iteration
24. Index i from step 1 is incremented to the next iteration, until st is accomplished.

**Algorithm 2.** Generating 2-scrolls using negative slopes

1   while i < st:

2    j = 0

3    j1 = 0

4    if x < B[0]:

5     PWL = m∗(x-B[0])+k[0])

6    while j < 2∗n-3:

7     if x >= B[j] and x < B[j+1]:

8      PWL = m[j]∗(x-B[j+1-j1])+k[j+1-j1]

9      if j == n-3:

10       j = j + 2

11       j1 = 1

12      else:

13       j = j + 1

14     if x >= B[n-2] and x < B[n-1]:

15      PWL = m[n-2]∗x

16     if x >= B[2∗n-3]:

17      PWL = m∗(x-B[2∗n-3])+k[2∗n-3]

18    xn.insert(i,x+h∗(alpha∗(y-x-PWL)))

19    yn.insert(i,y+h∗gamma∗(x-y+z))

20    zn.insert(i,z+h∗(-beta∗y))

21    x = xn[i]

22    y = yn[i]

23    z = zn[i]

24    i = i + 1

### 3.3 Oscillator based on sawtooth function

For Chua’s circuit based on sawtooth function, to generate even and odd number of scrolls from Eq (7), *f*(*x*) must be described as shown in Eqs (8) and (9), respectively. Algorithm 3 highlights the form in which the double-scroll chaotic attractor is simulated using Eq (8). The steps are executed as follows:

1. Loop to iterate st times
2. Verifies if x in *f*(*x*) is lower than the break point B[0]. If it is satisfied then
3. Variable PWL is equated to *m*(*x* − *B*[0]) + *k*[0], where *m* is the slope, B[0] the break point and *k*[0] the amplitude
4. Verifies if x in *f*(*x*) is higher than B[n-2], where n is the number of scrolls being generated. If it is satisfied then
5. PWL is equated to m∗(x-B[n-2])+k[2∗n-3], where m is the slope, B[n-2] the break point and k[2∗n-3] the amplitude
6. Loop to iterate n-2 times
7. Verifies if x in *f*(*x*) is between the break points B[j] and B[j+1]. If it is satisfied then
8. PWL is equated to m[j]∗(x-(B[j+1]+B[j])/2)
9. State variable x[n] is updated to x+h∗alpha(y-PWL)), where alpha is coefficient *α* in Eq (7), x and y are initial conditions, and h is the step size
10. State variable y[n] is updated to y+h∗gamma(x-y+z), where gamma is coefficient *γ* in Eq (7), and z the initial condition
11. State variable z[n] is updated to z+h(-beta∗y), where beta is coefficient *β* in Eq (7)
12. State variable x is updated to the next iteration
13. State variable y is updated to the next iteration
14. State variable z is updated to the next iteration
15. Index i from step 1 is incremented to the next iteration, until st is accomplished.

**Algorithm 3.** Generating 2-scrolls using sawtooth function

1   while i < st:

2    if x <= B[0]:

3     PWL = m∗(x-B[0])+k[0]

4    elif x > B[n-2]:

5     PWL = m∗(x-B[n-2])+k[2∗n-3]

6    for j in range(n-2):

7     if x > B[j] and x <= B[j+1]:

8      PWL = m[j]∗(x-(B[j+1]+B[j])/2)

9    xn.insert(i,x+h∗(alpha∗(y-PWL)))

10    yn.insert(i,y+h∗(x-y+z))

11    zn.insert(i,z+h∗(-beta∗y))

12    x = xn[i]

13    y = yn[i]

14    z = zn[i]

15    i = i+1

## 4 VHDL descriptions for the FPGA implementation of multi-scroll chaotic oscillators

From the pseudocodes listed above, one can infer the kind of digital hardware for generating chaotic behavior. For instance, to generate more than 2-scrolls or odd scrolls, one just needs to extend the PWL descriptions from Eqs (2), (3), (5), (6), (8) and (9), and all of them can be evaluated using comparators, as sketched in Algorithms 1, 2 and 3. This section shows the distribution of the digital word to establish the fixed-point format from high-level simulation using Python. Afterwards, our approach generates VHDL-code for the three multi-scroll chaotic oscillators detailed above. At the end, the generated VHDL-code is ready to be synthesized into an FPGA.

### 4.1 Fixed-point format for generating 2 and 30 scrolls

**Fig 4:**
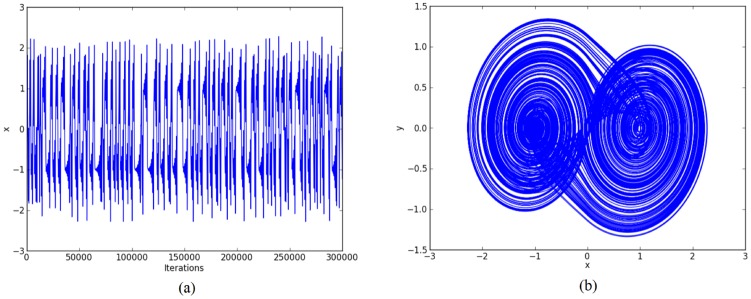
Double-scroll attractor using saturated function series. (a) State variable *x* and (b) Phase-space portrait *x* − *y*.

**Fig 5:**
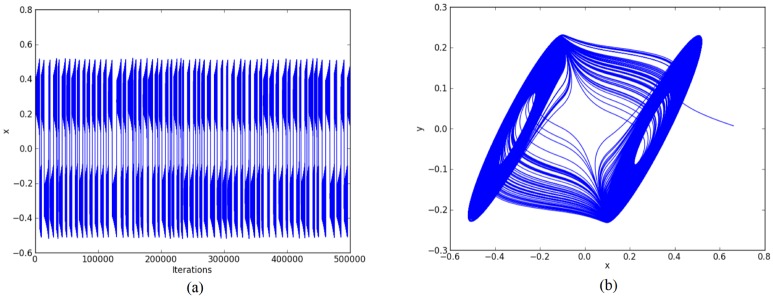
Double-scroll attractor using negative slopes. (a) State variable *x* and (b) Phase-space portrait *x* − *y*.

**Fig 6:**
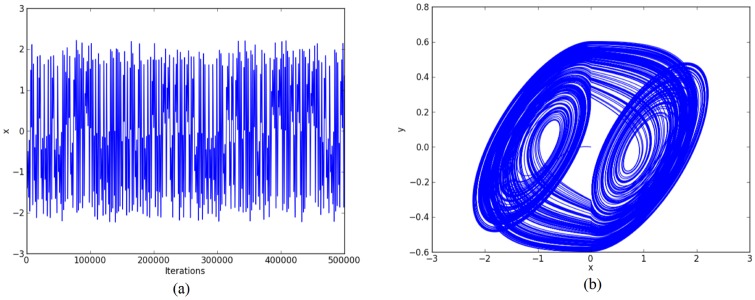
Double-scroll attractor using sawtooth function. (a) State variable *x* and (b) Phase-space portrait *x* − *y*.

To compute the fixed-point format being used for the VHDL descriptions, one needs to simulate the chaotic oscillator to know parameters like coefficient values and PWL characteristics, namely: break points, amplitudes, and slopes. Those parameter values for generating 2-scroll attractors for the three chaotic oscillators are the following:

- For the chaotic oscillator based on saturated function series: *k*_1_ = -1, *k*_2_ = 1, *B*_1_ = -0.0165, *B*_2_ = 0.0165, *a* = 0.7, *b* = 0.7, *c* = 0.7, and *d*_1_ = 0.7. The double-scroll attractor is shown in Fig 4.
- For Chua’s circuit using negative slopes: *m*_1_ = -0.276, *m*_2_ = -3.3036, *B*_1_ = -0.1, *B*_2_ = 0.1, *α* = 10, *β* = 15, *γ* = 1, *k*_1_ = 0.3036, *k*_2_ = -0.3036. The double-scroll attractor is shown in Fig 5.
- For Chua’s circuit using sawtooth function: *m* = 0.25, *B*_1_ = 0, *α* = 10, *β* = 16, *γ* = 1 and *k* = 0.25. The double-scroll attractor is shown in Fig 6.

**Fig 7:**
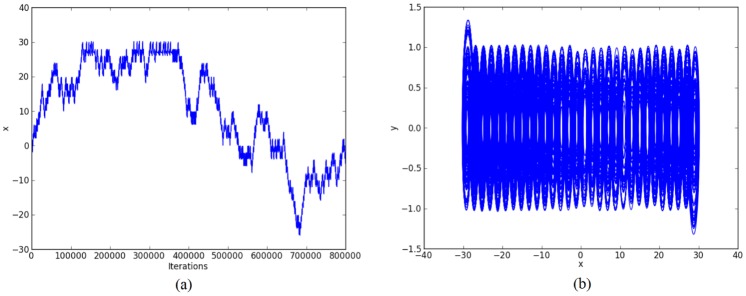
30-scroll attractor using saturated function series. (a) State variable *x* and (b) Phase-space portrait *x* − *y*.

**Fig 8:**
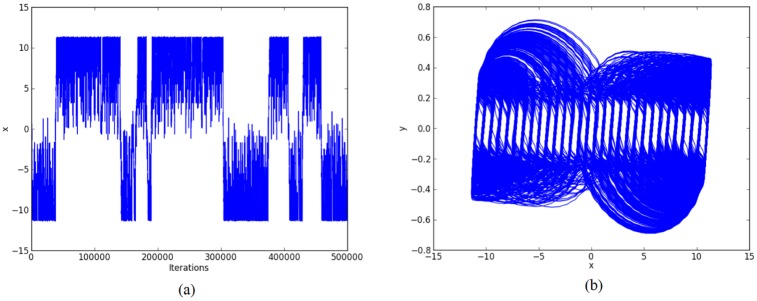
30-scroll attractor using negative slopes. (a) State variable *x* and (b) Phase-space portrait *x* − *y*.

**Fig 9:**
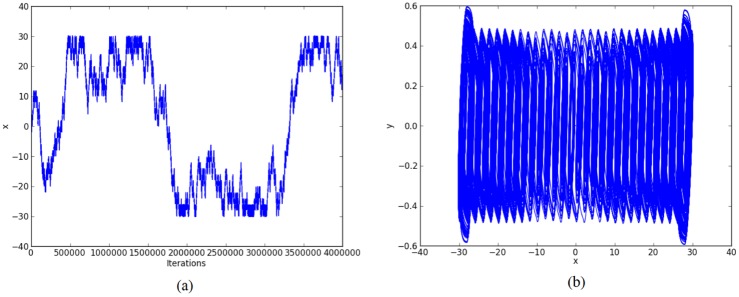
30-scroll attractor using sawtooth function. (a) State variable *x* and (b) Phase-space portrait *x* − *y*.

Other simulation results for generating 30-scrolls are shown in Figs 7–9.

From the simulation results, one can identify the ranges of the state variables and parameters that will serve to define the computer arithmetic to translate simulation parameters to VHDL-code. The binary digits will have integer and fractional parts and our algorithm converts real numbers to their 2’s complement. Basically, the integer part is converted through successive divisions and the fractional part with multiplications. From the 2’s complement numbers, one can generate VHDL code by interconnecting the required blocks that solve a discretized system of equations, e.g. Eqs (10), (11) and (12), as shown in the following subsections.

For example: Using 32 bits, the ranges of the state variables for generating 2-scrolls in Fig 4 are ±1.5 and ±3, in Fig 5 ±0.3 and ±0.6, and in Fig 6 ±0.6 and ±3. It is clear that 1 bit must be used for the sign, 2 bits for the integer part, and the rest for the fractional part, so that the fixed-point format can be established as 3.29.

Again, by using 32 bits, the ranges of the state variables for generating 30-scrolls in Fig 7 are ±1.5 and ±30, in Fig 8 ±0.8 and ±15, and in Fig 9 ±0.6 and ±30. Now, the number of bits for the integer part will be 5, so that the fixed-point format can be established as 6.26. Sometimes, this task on establishing the format also requires estimating the maximum value when multiplying 2 numbers.

### 4.2 On numerical methods, computer arithmetic and MLE

As one can infer, an important issue when implementing a dynamical system using fixed-point arithmetic is related to the necessary number of bits for the fractional part. The required number of bits can be estimated by trial and error techniques until observing the number of scrolls in the phase-space portrait, as already shown in [4, ch. 8]. So that one can reduce the number of bits for the fractional part until chaotic behavior remains. For instance, this subsection shows the reduction of bits for the fractional part to implement Chua’s chaotic oscillator with fixed-point arithmetic. It is obvious that one needs a metric to quantify chaotic behavior, therefore the three Lyapunov exponents are computed for different number of bits in the fractional part. The Lyapunov exponents give the most characteristic description of the presence of a deterministic non-periodic flow. They are asymptotic measures characterizing the average rate of growth (or shrinking) of small perturbations to the solutions of a dynamical system, and they provide quantitative measures of response sensitivity of a dynamical system to small changes in initial conditions [28]. That way, the goal is determining the minimum number of bits in the fractional part, when still the maximum Lyapunov exponent (MLE) remains in a similar value.

To measure the Lyapunov exponents, the initial state of the chaotic oscillator is set to

$$\begin{array}{ccc} y_{0} & \in & R^{12}\\ y_{0} & = & {\left[x_{0}^{\text{T}},e_{1}^{\text{T}},e_{2}^{\text{T}},e_{3}^{\text{T}}\right]}^{\text{T}} \end{array}$$

where [**e**_1_, **e**_2_, **e**_3_] = *I*, and *I* is the identity matrix of size 3 × 3. Thus, **e**_*i*_, for *i* = 1, 2, 3, are each unitary column vector of the identity matrix *I*.

The original system described by Eq (4) is observed by expanding it with other three systems. If $x={\left[\dot{x},\dot{y},\dot{z}\right]}^{\text{T}}$ represent one state of the original dynamical system at any *t* > 0, then the states in the three new observational systems will be **y** = [**x**, **x**_1_, **x**_2_, **x**_3_]^T^. The observational system is integrated by several steps until an orthonormalization period *T*_*O*_ is reached. After this, the state of the variational system is orthonormalized by using the standard Gram-Schmidt method. The next integration is carried out by using the new orthonormalized vectors as initial conditions [28].

The Lyapunov exponents measure the long time sensitivity of the flow in **x** with respect to the initial data **x**_0_ at the directions of every orthogonalized vector. This measure is taken when the variational system is orthonormalized. In this manner, if **y** = [**x**, **p**_1_, **p**_2_, **p**_3_]^T^ is the state after the matrix [**x**_1_, **x**_2_, **x**_3_] is orthonormalized, then the Lyapunov exponent *λ*_*i*_, for *i* = 1, 2, 3 is evaluated by

$$\begin{array}{c} \lambda_{i}\approx \frac{1}{T}\sum_{j=T_{O}}^{T}\ln∥p_{i}∥ \end{array}$$ (13)

In this work the simulation of the expanded system was carried on with fixed integer arithmetic, using Forward Euler method with a time-step of 0.001. *T*_*O*_ was set to 5 seconds and the period *T* to 2000 seconds, respectively. The number of bits for the integer part was established by looking at the phase-space portraits of the expanded system, so that 8-bits are good enough for the integer part. For the fractional part, we test the behavior by using from 10 to 30 bits, therefore, Table 1 lists the values of the Lyapunov exponents computed with formats of 9.10 to 9.30.

**Table 1:** Lyapunov exponents for Chua’s chaotic oscillator computed with integer arithmetic and with formats 9.10 to 9.30. f.p.n. indicates Lyapunov exponents computed with floating point numbers.

Bits in the fractional part	Lyapunov exponents
f.p.n.	0.061775 -0.000225 -2.242316
30	0.065421 0.000175 -2.216514
29	0.066633 0.000175 -2.213263
28	0.065321 0.000046 -2.217932
27	0.067895 0.000074 -2.211247
26	0.066553 0.000082 -2.216548
25	0.066436 -0.000094 -2.214181
24	0.065620 0.000032 -2.213422
23	0.065213 0.000108 -2.215123
22	0.064137 0.000126 -2.221193
21	0.064985 0.000080 -2.218178
20	0.064406 0.000039 -2.217326
19	0.064579 -0.000064 -2.218593
18	0.066106 0.000220 -2.214908
17	0.066738 0.000249 -2.212879
16	0.063966 -0.000027 -2.198159
15	0.065293 0.000086 -2.159459
14	0.063845 0.000222 -2.162861
13	0.058283 0.001086 -2.186606
12	0.011525 0.015968 -2.271788
11	0.006752 0.016839 -2.272423
10	0.037251 0.012891 -2.320074

**Fig 10:**
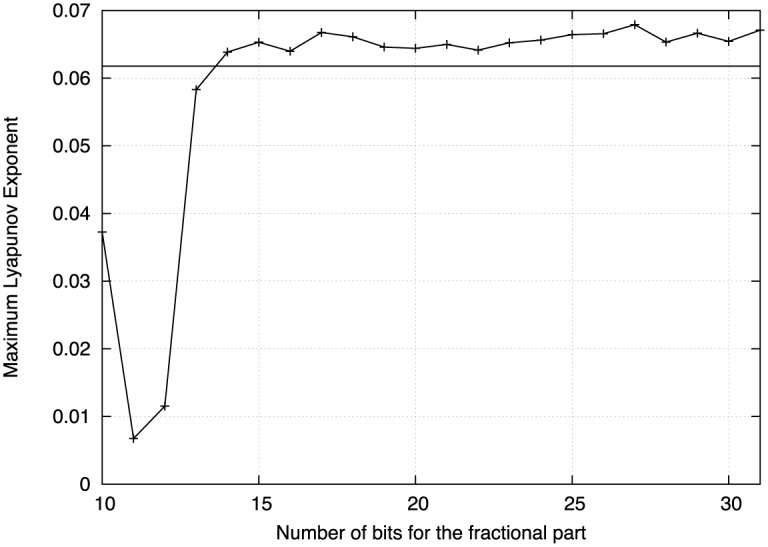
Maximum Lyapunov exponent (MLE) against number of bits in the fractional part. The horizontal line marks MLE computed with floating point numbers (f.p.n.).

The computation of the Lyapunov exponents using Eq (13) was performed using floating point numbers, by applying the fourth-order Runge-Kutta method with a time step of 0.01, and whose result are listed in the first row of Table 1. The remaining values in that Table were computed using fixed integer arithmetic and Forward Euler with a time-step of 0.001. Fig 10 shows the maximum Lyapunov exponent (MLE) against the number of bits for the fractional part. The value computed by applying the fourth-order Runge-Kutta method is shown by the horizontal line at 0.061775, so that from these results, it is clear that by applying Forward Euler and fourth-order Runge-Kutta one gets similar values of the MLE. In addition, discretizing the ordinary differential equations with Forward Euler will require the lowest number of FPGA resources. Finally, one can conclude that for a hardware realization, at least 14 bits for the fractional part are required to keep the chaotic oscillator under a similar MLE value.

### 4.3 Block description to generate the hardware associated to discretized equations

**Fig 11:**
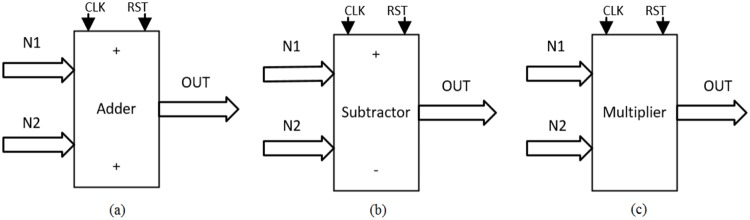
Basic building blocks for VHDL programming.

**Fig 12:**
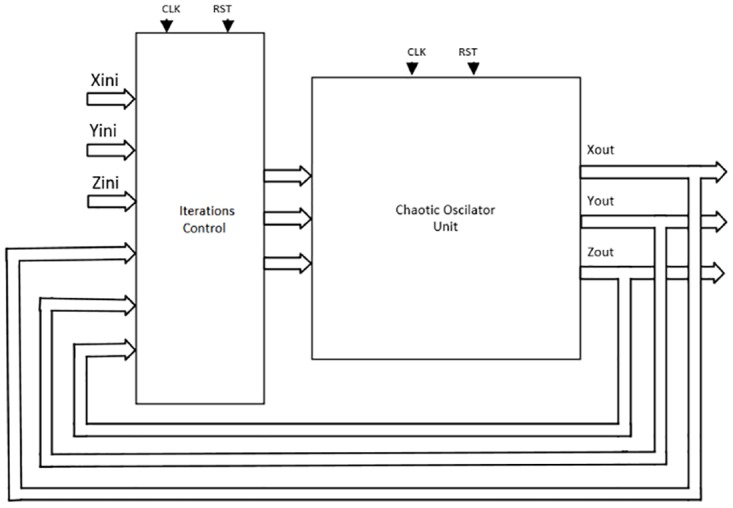
High-level hardware interconnection for the multi-scroll chaotic oscillators.

As detailed in [4, 21], the solution to the system of differential equations modeling a chaotic oscillator like Eqs (1), (4) and (7), needs the application of a numerical method to obtain their discretized descriptions like in Eqs (10)–(12), respectively. From those equations one can identify digital blocks like comparators for implementing the PWL functions, adders, subtractors and multipliers. Each block will process bits according to the selected format. For instance, Fig 11 shows the description of the adder, subtractor and multiplier. They are synchronous to take control on the iterative process and have associated a delay of a clock pulse CLK. In this manner, the time propagation from the input to the output in the chaotic oscillator unit shown in Fig 12, equals the maximum number of series-connected blocks. In Fig 12 one can identify the block Iterations control, which embeds a timer and a multiplexer to control the iterations during the numerical integration, i.e. st listed in the pseudocodes in Sect. 3. It also processes the initial conditions from the first iteration loop.

**Fig 13:**
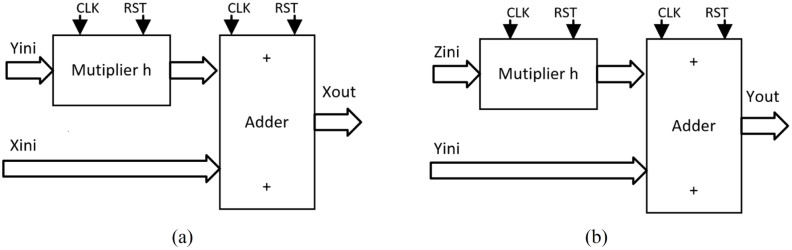
Hardware connection for implementing. (a) *x*[*k* + 1] = *x*[*k*] + *hy*[*k*], and (b) *y*[*k* + 1] = *y*[*k*] + *hz*[*k*] from Eq (10).

**Fig 14:**
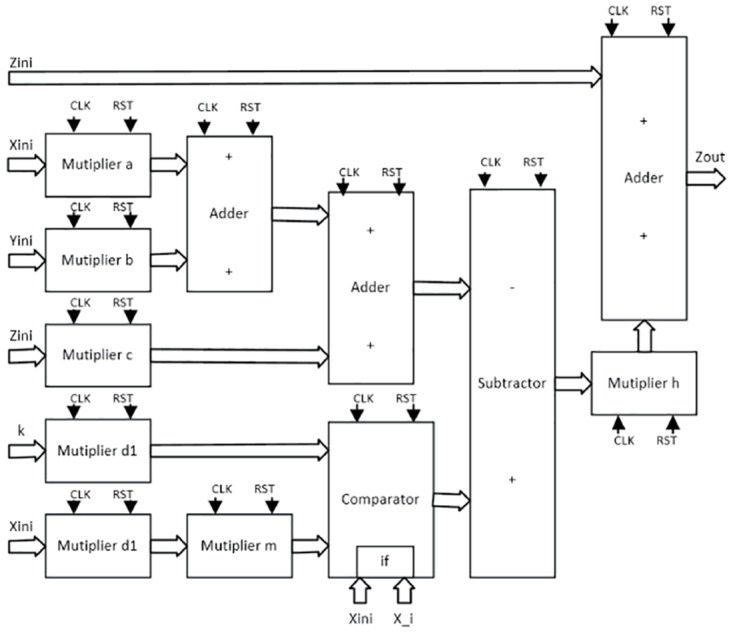
Hardware connection for implementing. *z*[*k* + 1] = *z*[*k*] + *h*(−*ax*[*k*] − *by*[*k*] − *cz*[*k*] + *d*_1_ *f*(*x*[*k*])) from Eq (10).

From the discretized equations of the oscillator based on saturated functions in Eq (10), the state variables *x* and *y* are easy to implement as shown in Fig 13. However, *z* needs more hardware, as shown in Fig 14. In this manner, its propagation time is the largest and it controls the iteration loop in Fig 12. For this chaotic oscillator, the number of building blocks required in the Chaotic Oscillator Unit from Fig 12, can be calculated as given in Table 2, where n is the number of scrolls being generated.

**Table 2:** Estimation of required building blocks for the multi-scroll chaotic oscillators.

Block	Saturated functions	Negative slopes	Sawtooth
Adders	3(n-2)+8	3n+2	n+4
Subtractors	n	2n+1	n+2
Multipliers	5(n-3)+12	2n+5	2n+4

**Fig 15:**
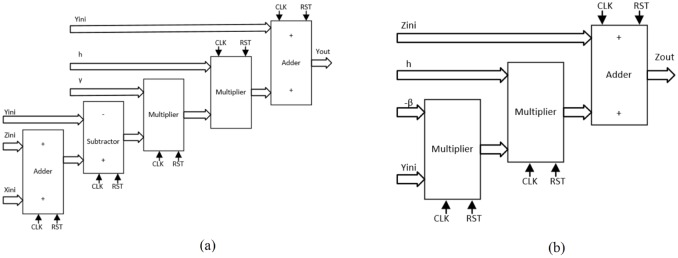
Hardware connection for implementing. (a) *y*[*k* + 1] and (b) *z*[*k* + 1] from Eqs (11) and (12).

**Fig 16:**
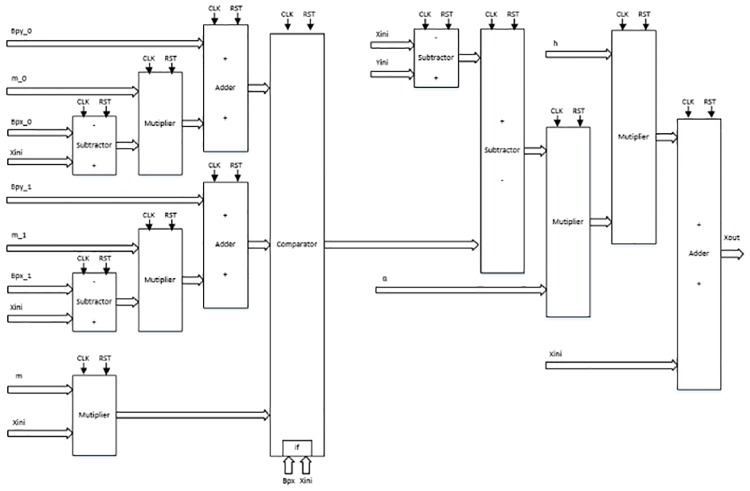
Hardware connection for implementing *x*[*k* + 1] from Eq (11).

**Fig 17:**
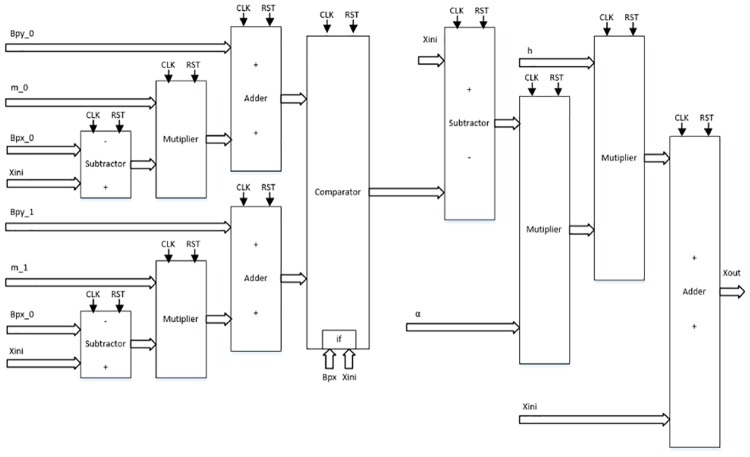
Hardware connection for implementing *x*[*k* + 1] from Eq (12).

Chua’s chaotic oscillators have similar equations, the main difference is the evaluation of state variable *x* and their corresponding PWL functions for negative slopes and sawtooth. As state variables *y* and *z* have the same description from Eqs (11) and (12), Fig 15 shows their implementations. For *x*, the hardware realization using negative slopes is shown in Fig 16, and using sawtooth function is shown in Fig 17.

Again, for both Chua’s chaotic oscillators based on negative slopes and sawtooth function, the number of building blocks required in the Chaotic Oscillator Unit from Fig 12, can be calculated as given by Table 2. These resources estimations are just the ones required to solve the discretized equations, and they can be incremented according to the length of the digital word that depends on the selected format.

### 4.4 VHDL-code generation

VHDL is the acronym of *Very High-Speed Integrated Circuit Hardware Description Language*, it was developed around 1980 at the request of the U.S. Department of Defense. At the beginning, the main goal of VHDL was the electric circuit simulation; however, tools for synthesis and implementation in hardware based on VHDL behavior or structure description files were developed later. With the increasing use of VHDL, the need for standardized was generated. In 1986, the Institute of Electrical and Electronics Engineers (IEEE) standardized the first hardware description language through the 1076 and 1164 standards. Nowadays, VHDL is technology/vendor independent, then VHDL codes are portable and reusable.

In Subsection 4.1 one can see the main parameters of a 2-scroll chaotic oscillator, like: coefficient values, break points, amplitudes, and slopes. They are used to perform a high-level simulation to identify the ranges of the state variables and iteration parameters that serve to define the computer arithmetic to translate simulation parameters to VHDL-code. Our approach can automatically generate VHDL code by interconnecting the required blocks that solve a discretized system of equations, e.g. Eqs (10)–(12), as already shown in Subsection 4.3. In this manner, this subsection shows the VHDL-code associated to the blocks required to synthesize the three multi-scroll chaotic oscillators detailed in Sect. 2. For instance, the main steps in generating VHDL-code can be summarized as follows:

Step 1: Select the number of scrolls being generated, coefficient values and characteristics of the PWL function of the desired multi-scroll chaotic oscillator. The break points are directly related to the number of scrolls and must be provided from the left (the most negative) to the right (the most positive), according to Figs 1, 2 or 3. Similarly, the saturation levels in Fig 1, or amplitudes in Figs 2 or 3 must be provided from the bottom (most negative) to the top (most positive).

Step 2: The high-level simulation is performed and our approach verifies the number of desired scrolls. From simulation data, the fixed-point format is established, as discussed in Subsection 4.1, where the ranges in Figs 4–6, leads us to use format 3.29 (using 32 bits), and from Figs 7–9, the ranges require a format 6.26. After the format is established, our approach connects the required blocks as detailed in subsection 4.3.

Step 3: Our approach creates a file containing the libraries and code for the blocks implementing Fig 12, where the block Iterations Control embeds a counter and a multiplexer to take control on the iterative process. In our examples, the counter provides a delay of 8 clock cycles CLK to process all signals at each iteration. The block Chaotic Oscillator Unit embeds all blocks implementing the desired multi-scroll chaotic oscillator.

The VHDL-codes for all the required blocks are shown in the following Algorithms. As one sees, they are described using 28 bits as the word length. This is not an issue, since our approach can generate the codes automatically. The signals are also described for each case.

The blocks multiplier, adder and subtractor have the following signal descriptions:

- CLK: This is the master clock of the FPGA. All the operations are performed based on the clock frequency.
- RST: Reset of the system, puts the outputs to zero. Restarts the system.
- N1 and N2: Input data.
- OUTS: Output data.
- sena1: Internal signal with the double of bits from the word length to perform the multiplication.

**Algorithm 4.** Multiplier in VDHL

1   entity multiplier is

2   port(

3    CLK: in std_logic;

4    RST: in std_logic;

5    N1: in std_logic_vector(27 downto 0);

6    N2: in std_logic_vector(27 downto 0);

7    OUTS: out std_logic_vector(27 downto 0) := (others=>‘0’));

8   end multiplier;

9   architecture code of multiplier is

10   signal sena1: signed(55 downto 0) := (others=>‘0’);

11   begin

12   process(CLK,RST)

13   begin

14    if RST = ‘1’ then

15     OUTS <= (others => ‘0’);

16    elsif rising_edge(CLK) then

17     sena1 <= (signed(N1)∗signed(N2));

18     OUTS <= std_logic_vector(sena1(51 downto 24));

19    end if;

20   end process;

21   end code;

**Algorithm 5.** Adder in VHDL

1   entity adder is

2   port(

3    CLK: in std_logic;

4    RST: in std_logic;

5    N1: in std_logic_vector(27 downto 0);

6    N2: in std_logic_vector(27 downto 0);

7    OUTS: out std_logic_vector(27 downto 0) := (others => ‘0’));

8   end adder;

9   architecture code of adder is

10   begin

11   process(CLK,RST)

12   begin

13    if RST = ‘1’ then

14     OUTS <= (others => ‘0’);

15    elsif rising_edge(CLK) then

16     OUTS <= std_logic_vector(signed(N1) + signed(N2));

17    end if;

18   end process;

19   end code;

**Algorithm 6.** Subtractor in VHDL

1   entity subtractor is

2   port(

3    CLK: in std_logic;

4    RST: in std_logic;

5    N1: in std_logic_vector(27 downto 0);

6    N2: in std_logic_vector(27 downto 0);

7    OUTS: out std_logic_vector(27 downto 0) := (others => ‘0’));

8   end subctractor;

9   architecture code of subtractor is

10   begin

11   process(CLK,RST,N1,N2)

12   begin

13    if RST = ‘1’ then

14     OUTS <= (others => ‘0’);

15    elsif rising_edge(CLK) then

16     OUTS <= std_logic_vector(signed(N1) − signed(N2));

17    end if;

18   end process;

19   end code;

**Algorithm 7.** Iterations control block in VHDL

1   entity IterCtrl is

2   port(

3    CLK: in std_logic;

4    RST: in std_logic;

5    Xini: in std_logic_vector(27 downto 0);

6    Yini: in std_logic_vector(27 downto 0);

7    Zini: in std_logic_vector(27 downto 0);

8    Xout: out std_logic_vector(27 downto 0) := (others => ‘0’);

9    Yout: out std_logic_vector(27 downto 0) := (others => ‘0’);

10    Zout: out std_logic_vector(27 downto 0) := (others => ‘0’));

11   end IterCtrl;

12   architecture code of IterCtrl is

13   signal dz: std_logic_vector(27 downto 0) := “0000000000000000000000000000”;

14   signal dy: std_logic_vector(27 downto 0) := “0000000000000000000000000000”;

15   signal dx: std_logic_vector(27 downto 0) := “0000000101100110011001100110”;

16   begin

17   process(CLK,RST)

18   variable count: integer := 0;

19   begin

20    if RST = ‘1’ then

21     dx <= (others => ‘0’);

22     dy <= (others => ‘0’);

23     dz <= (others => ‘0’);

24    elsif rising_edge(CLK) then

25     if count = 8 then

26      count := 0;

27      dx <= Xini;

28      dy <= Yini;

29      dz <= Zini;

30     else

31      count := count +1;

32     end if;

33    end if;

34   end process;

35   Xout <= dx;

36   Yout <= dy;

37   Zout <= dz;

38   end code;

Iterations control block (Algorithm 7) has the signals:

- CLK and RST are the same for all blocks.
- Xini, Yini and Zini: Feeds input signals to the Chaotic Oscillator Unit, until accomplishing st iterations as sketched in Sect. 3.
- Xout, Yout and Zout: Output data that will be processed by the Iterations control block to perform st iterations.
- dx, dy and dz: Initial conditions for each iteration. These registers save the output data of the state variables and then feeds them as input signals Xini, Yini and Zini.
- count: Signal to update the initial values to the Chaotic Oscillator Unit at each iteration. This signal is enabled after 8 clock cycles (CLKs), which is the time required by the maximum number of series-connected blocks.

**Algorithm 8.** Comparator for implementing saturated functions in VHDL

1   entity comparator is

2   port(

3    CLK: in std_logic;

4    RST: in std_logic;

5    dato_sat0: in std_logic_vector(27 downto 0);

6    dato_sat1: in std_logic_vector(27 downto 0);

7    dato_pen0: in std_logic_vector(27 downto 0);

8    dato_X: in std_logic_vector(27 downto 0);

9    dato_S: out std_logic_vector(27 downto 0) := (others => ‘0’));

10   end comparator;

11   architecture code of comparator is

12   constant B0: std_logic_vector(27 downto 0) := “1000000000000000000010101000”;

13   constant B1: std_logic_vector(27 downto 0) := “1111111110111100011010101000”;

14   constant B2: std_logic_vector(27 downto 0) := “0000000001000011100101011000”;

15   constant B3: std_logic_vector(27 downto 0) := “0111111111111111111111101111”;

16   begin

17   process(CLK,RST,dato_X)

18   begin

19    if RST = ’1’ then

20     dato_S<= (others => ‘0’);

21    elsif rising_edge(CLK) then

22     if dato_X > B0 AND dato_X < B1 then

23      dato_S <= dato_sat0;

24     elsif dato_X > B2 AND dato_X < B3 then

25      dato_S <= dato_sat1;

26     else

27      dato_S <= dato_pen0;

28     end if;

29    end if;

30   end process;

31   end code;

The PWL functions are implemented using comparators. If the PWL segments increase, as detailed in Sect. 3, then also the number of comparisons do. The comparator blocks have CLK and RST signals as the previous blocks.

The PWL function for the oscillator based on saturated functions is implemented by Algorithm 8, where:

- dato_sat0: Input data for the first level of saturation.
- dato_sat1: Input data for the second level of saturation.
- dato_pen0: Input data for the slope.
- dato_X: Input data of the state variable *x* to perform comparisons.
- dato_S: Output data that takes the value from one input like dato_sat0, dato_sat1, or dato_pen0.
- B0, B1, B2 and B3: Constants to represent the break points from the left to the right of the PWL functions.

The PWL function for the oscillator based on negative slopes is implemented by Algorithm 9, where:

- dato_pen0: Input data for the first slope located on the left of the PWL function.
- dato_pen1: Input data for the last slope, which is the same as the first.
- dato_penm: Input data for the central slope.
- dato_X: Input data of the state variable *x* to perform comparisons.
- dato_S: Output data that takes the value from one input like dato_pen0, dato_pen,1 or dato_penm.
- B0 and B1: Constants to represent the break points from the left to the right of the PWL functions.

**Algorithm 9.** Comparator for implementing negative slopes in VHDL

1   entity comparador is

2   port(

3    CLK: in std_logic;

4    RST: in std_logic;

5    dato_pen0: in std_logic_vector(27 downto 0);

6    dato_pen1: in std_logic_vector(27 downto 0);

7    dato_X: in std_logic_vector(27 downto 0);

8    dato_S: out std_logic_vector(27 downto 0) := (others => ‘0’));

9   end comparador;

10   architecture complicado of comparador is

11   constant B0: std_logic_vector(27 downto 0) := (others => ‘0’)

12   begin

13   process(CLK,RST,dato_X)

14   begin

15    if RST = ’1’ then

16     dato_S<= (others => ‘0’);

17    elsif rising_edge(CLK) then

18     if dato_X < B0 then

19      dato_S <= dato_pen0;

20     else

21      dato_S <= dato_pen1;

22     end if;

23    end if;

24   end process;

25   end complicado;

**Algorithm 10.** Comparator for implementing sawtooth function in VHDL

1   entity comparator is

2   port(

3    CLK: in std_logic;

4    RST: in std_logic;

5    dato_pen0: in std_logic_vector(27 downto 0);

6    dato_pen1: in std_logic_vector(27 downto 0);

7    dato_penm: in std_logic_vector(27 downto 0);

8    dato_X: in std_logic_vector(27 downto 0);

9    dato_S: out std_logic_vector(27 downto 0) := (others => ‘0’));

10   end comparator;

11   architecture complicado of comparador is

12   constant B0: std_logic_vector(27 downto 0) := “1111111100110011001100110100”;

13   constant B1: std_logic_vector(27 downto 0) := “0000000011001100110011001100”;

14   begin

15   process(CLK,RST,dato_X)

16   begin

17    if RST = ’1’ then

18     dato_S<= (others => ‘0’);

19    elsif rising_edge(CLK) then

20     if dato_X < B0 then

21      dato_S <= dato_pen0;

22     elsif dato_X >= B1 then

23      dato_S <= dato_pen1;

24     else

25      dato_S <= dato_penm;

26     end if;

27    end if;

28   end process;

29   end complicado;

The PWL function for the oscillator based on sawtooth function is implemented by Algorithm 10, and it is similar as the last two comparators, where:

- dato_pen0: Input data for the first slope.
- dato_pen1: Input data for the last slope, which is the same as the first.
- dato_X: Input data of the state variable *x* to perform comparisons.
- dato_S: Output data that takes the value from one input like dato_pen0, dato_pen1.
- B0: Constant to represent the break point.

The generated VHDL-code is ready to be synthesized into an FPGA, so that the following Section shows experimental results.

## 5 Experimental results

**Fig 18:**
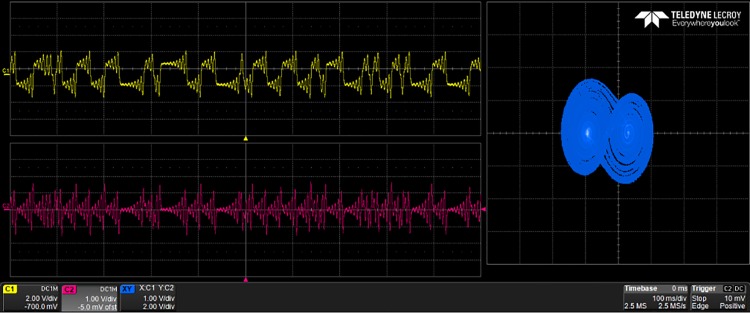
2-scroll attractor using saturated function series.

**Fig 19:**
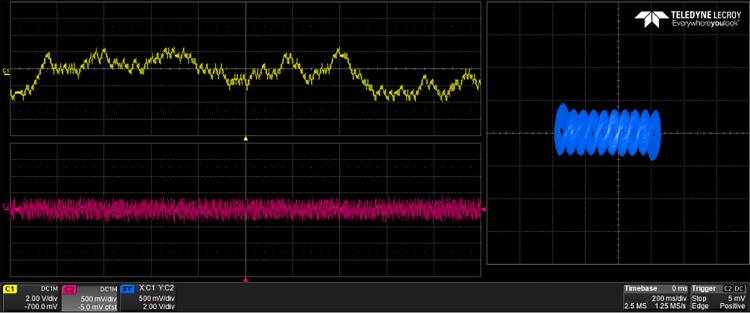
10-scroll attractor using saturated function series.

**Fig 20:**
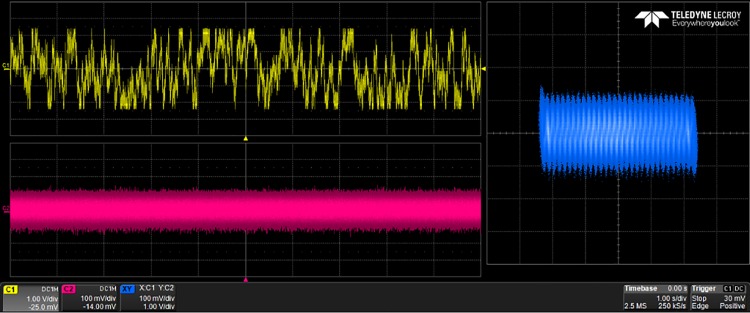
30-scroll attractor using saturated function series.

**Fig 21:**
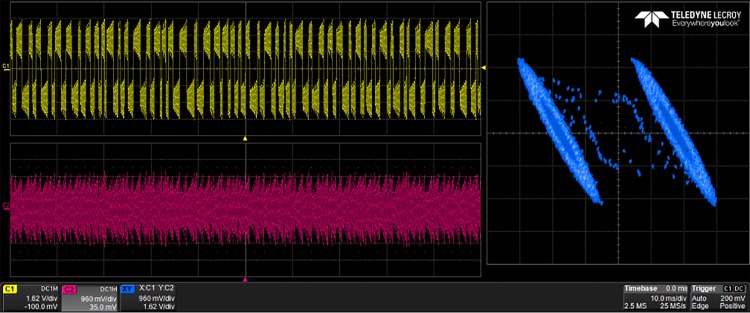
2-scroll attractor using negative slopes.

**Fig 22:**
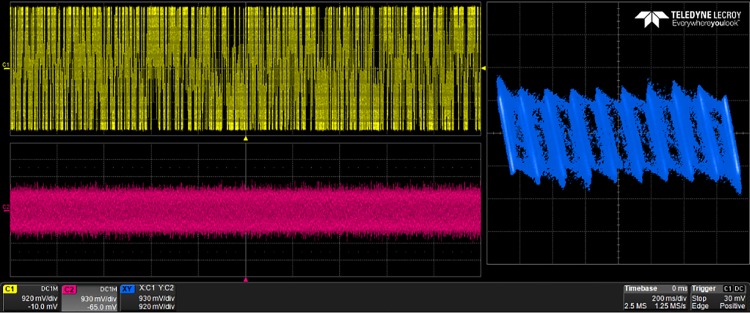
10-scroll attractor using negative slopes.

**Fig 23:**
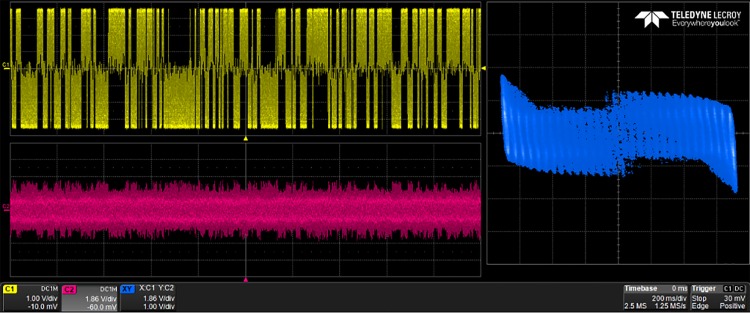
30-scroll attractor using negative slopes.

**Fig 24:**
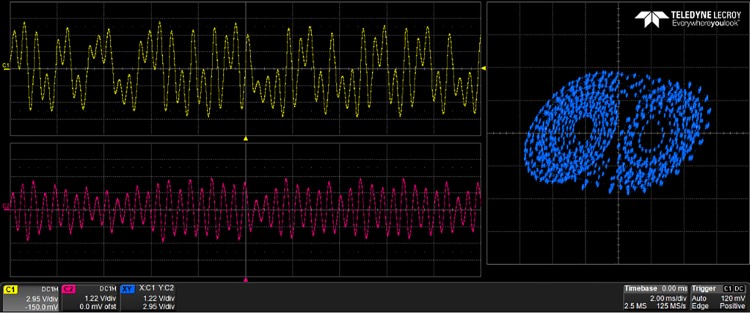
2-scroll attractor using sawtooth function.

**Fig 25:**
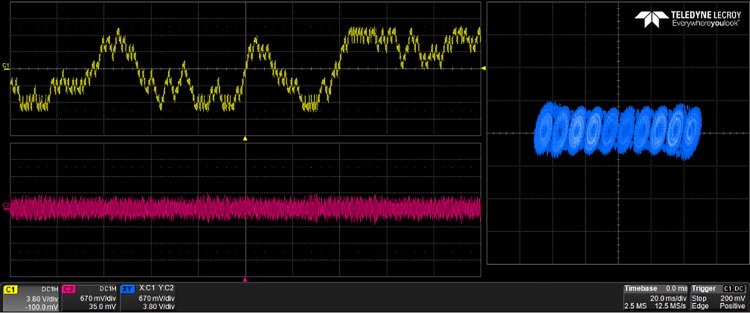
10-scroll attractor using sawtooth function.

**Fig 26:**
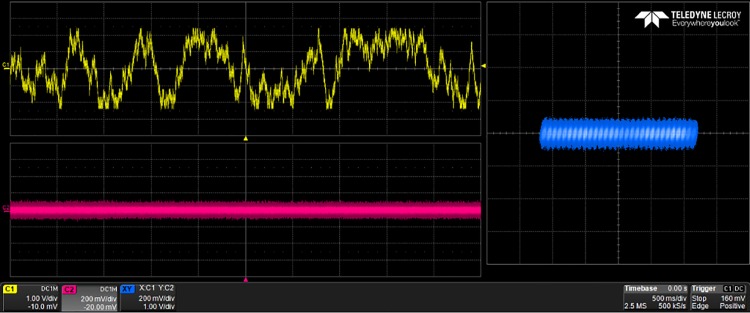
30-scroll attractor using sawtooth function.

The generated VHDL-codes for the synthesis of multi-scroll chaotic oscillators based on PWL functions were implemented in the Altera’s FPGA EP4CGX150DF31C7 Cyclone IV GX. The used resources are listed in Table 3, and the experimental attractors are shown in Figs 18–26, where one can appreciate the good agreement with simulation results from Sect. 4. In all those figures the state variable *x* is shown on the top left-side, *y* on the bottom left-side, and the phase-space portrait *x* − *y* on the right-side. One can count the number of scrolls from the phase-space portraits, and from Lyapunov exponents evaluation, one can infer that the more scrolls are generated the more complex behavior. In this manner, and from the experimental results, it is clear that engineering applications like in [4] can be realized in a very short time. This is the advantage of FPGAs for fast prototyping, and we have introduced a Python-based approach for the generation of VHDL-code that is ready for FPGA synthesis.

**Table 3:** Resources for generating 2, 10 and 30-scroll chaotic attractors using fixed-point format with 6.22 bits.

Oscillator	Scrolls	Multipliers 9-bits	Logic elements	Registers
Saturated Functions	2	73	1030	760
	10	79	2045	1386
	30	160	4354	2870
Chua Negative Slopes	2	44	902	776
	10	172	3767	1953
	30	492	10747	5342
Chua Sawtooth	2	44	893	623
	10	168	3023	1886
	30	470	10354	4870

## 6 Conclusions

We have introduced an approach programmed in Python for the generation of VHDL-code associated to multi-scroll chaotic oscillators that are based on PWL functions. The pseudocodes for simulating three kinds of chaotic oscillators were listed to infer the FPGA implementation of the PWL functions based on saturated functions series, negative slopes and sawtooth one. From the high-level simulation, our algorithm determines the fixed-point format to interconnect digital blocks associated to the discretized equations of the chaotic oscillators. The PWL functions are then implemented by using comparator blocks that can increase the number of comparisons according to the number of scrolls being generated. It was highlighted that these tasks are performed from the description of the dynamical equations and PWL functions, to the interconnection of the digital blocks and generation of the VHDL-code, which is portable, reusable and open source to be synthesized in an FPGA of any vendor.

It is worthy mentioning that the FPGA resources depend on the discretization approach. For instance, Subsection 4.2 showed that Forward Euler and the fourth-order Runge-Kutta methods computed similar values of the Lyapunov exponents, and also when using floating point and fixed integer arithmetic. However, the number of bits for the fractional part matters. As shown in Table 1, at least 14 bits for the fractional part are required to keep the chaotic oscillator under a similar MLE value.

Finally, it can be concluded that our approach for generating VHDL descriptions can estimate the number of required blocks from the equations listed in Table 2, which depend on the number of scrolls being generated. And, among the three multi-scroll chaotic oscillators that were implemented into an FPGA, we can conclude that the one based on saturated function series requires lower number of hardware resources, as showed by Table 3.
